# Lifelong recurrent takotsubo cardiomyopathy: a case report

**DOI:** 10.1093/ehjcr/ytz191

**Published:** 2019-10-24

**Authors:** Shekinah Chandy, Dana K Dawson

**Affiliations:** 1 Department of Cardiology, Aberdeen Royal Infirmary, Foresterhill Campus, Aberdeen AB25 2ZN, UK; 2 School of Medicine and Dentistry, University of Aberdeen, Aberdeen AB25 2ZD, UK

**Keywords:** Takotsubo cardiomyopathy, Lifelong recurrence, Variant morphologies, Acute myocardial infarction, Case report

## Abstract

**Background:**

Takotsubo cardiomyopathy is a transient left ventricular dysfunction with an established recurrence rate in populations, however, recurrences in the same individual have not been well described.

**Case summary:**

We present a 76-year-old woman who had likely a total of six recurrent takotsubo cardiomyopathy episodes spanning over 33 years. Her diagnosis of takotsubo cardiomyopathy was first made in 2014 when she presented with chest pain, raised cardiac enzymes, and the presence of normal coronary arteries. Cardiac magnetic resonance was performed, ruling out any current or previous myocardial infarction. Subsequently, she had two further recurrences in 2015 and 2018. Stressors were identified on three occasions. She was diagnosed with ‘myocardial infarction’ in 1986, 1988, and 1998 when she presented with chest pain and electrocardiogram changes, despite demonstrating normal coronary arteries on each occasion.

**Discussion:**

This case demonstrates three confirmed recurrent episodes of takotsubo in the same individual, showing three different left ventricular phenotypic morphologies on the background of three previous episodes of ‘myocardial infarction with normal coronary arteries’, which most likely might have been takotsubo episodes as well. Any myocardial infarction-type injury was definitely ruled out in the 2014 admission instigating a potential change in this patient’s past medical history and implicitly requirement for lifelong secondary prevention. It is notably difficult to make a confirmed diagnosis of takotsubo cardiomyopathy back in 1986, 1988, and 1998 due to the lack of awareness in this novel topic.

**Learning points**

- It is important to distinguish between acute myocardial infarction and takotsubo cardiomyopathy as this will dictate the patient’s requirement for secondary prevention and counselling regarding the aftermath of the two different conditions.
- Clinicians should be aware of the possibility of recurrent episodes of takotsubo cardiomyopathy in the same individual with multiple variant morphologies.

## Introduction

Takotsubo cardiomyopathy mimics acute myocardial infarction in its presentation and has a recognized recurrence rate.^1^^,^^2^ So far, the literature focused on the re-occurrence of the syndrome in populations without particular attention to the number of recurrences in a certain individual. We, hereby, present a case of a patient having three confirmed recorded recurrences at the same institution on the background of another three previous (historic) admissions at the same institution, which most likely represented takotsubo episodes but mislabelled as ‘myocardial infarction’ according to the contemporaneous knowledge at the time. She had preserved ejection fraction after each recovery.

## Timeline 

**Table:** 

Time	Events
1986	Presented with chest pain. Electrocardiogram (ECG) showed widespread anterolateral T-wave inversion and prolonged QTc. Angiogram revealed normal coronary arteries.
1988	Represented similarly with chest pain and similar electrocardiographic changes as 1986.
1998	Admitted with chest discomfort. Elevated creatine kinase to 233. Electrocardiogram showed similar changes as 1986. Normal left and right coronary arteries.
January 2014	Presented with chest pain following recent bereavement of neighbour. Raised cardiac markers and normal coronary arteries. Electrocardiogram showed obvious hyperacute T wave in anterior leads. Apical ballooning noted on echocardiogram. Cardiac magnetic resonance imaging (MRI) with late gadolinium enhancement ruled out myocardial infarction.
June 2015	With ongoing stress at home, she once again presented with chest pain, ECG showed widespread hyperacute T waves in anterolateral leads, raised cardiac enzymes, and normal coronaries.
June 2018	Represented with chest pain, ECG showed T-wave inversion in lateral leads and prolonged QTc, with no known precipitating events. Cardiac MRI demonstrate focal lateral wall ballooning.

## Case presentation 

**Figure 1:**
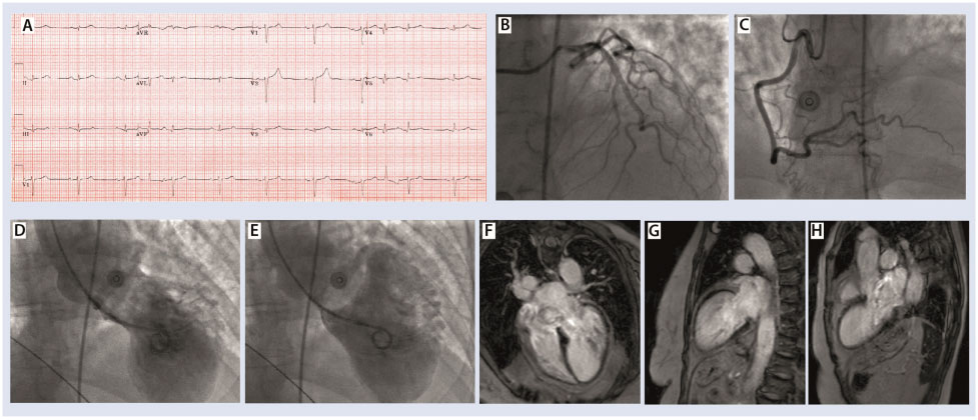
(*A*) Presenting electrocardiogram. (*B* and *C*) Left and right coronary arteries, respectively, (*D* and *E*) End-systolic and end-diastolic frames of the left ventriculogram demonstrating extensive apical ballooning. (*F*–*H*) Late gadolinium enhancement ruling out any myocardial infarction.

A 76-year-old ex-smoker White Scottish woman first presented in 2014 via Emergency Department, with central chest pain in the context of recent bereavement. Her admission troponin was increased to 8.6 times the upper limit of normal contemporary range. Her B-type natriuretic peptide was 1220 pg/mL. All other laboratory test were unremarkable. Her admission electrocardiogram (ECG) showed in *Figure 1A*. Coronary angiography revealed normal coronary arteries (*Figure 1B* and *C*) but the left ventriculogram showed extensive apical ballooning (*Figure 1D*–*E*). An echocardiogram demonstrated apical ballooning (Supplementary material online, *Movie M4*). Cardiac magnetic resonance imaging (MRI) ruled out a myocardial infarction (*Figure 1F*–*H*). Her left ventricular ejection fraction recovered entirely after 4 months and she had a classical diagnosis of apical takostubo. Her InterTak diagnostic score was 61, with 58.6% probability of takotsubo.

**Figure 2:**
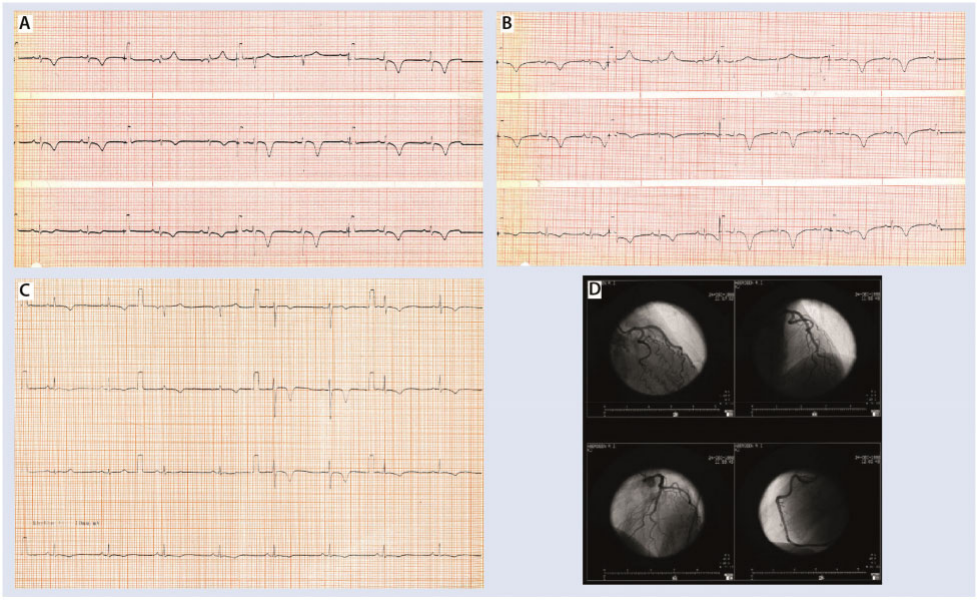
(*A–C*) Presenting electrocardiograms in 1986, 1988, and 1998, respectively. (*D)* Digitalized left and right coronary arteries in 1998.

The patient had a significant family history of ischaemic heart disease with sister and brother dying of myocardial infarction in their 6th decade of life. Her past medical history included hypercholesterolaemia and three previous episodes which were labelled ‘myocardial infarctions’ in 1986, 1988, and 1998. These historic presenting ECG’s are shown in *Figure 2A*–*C*, respectively. Her cardiac enzymes in 1986 and 1988 were unremarkable but in 1998 her initial creatine kinase was raised to 1.7 times the upper limit of normal contemporary range. She underwent coronary angiography which demonstrated normal coronary arteries on each occasion. Digitized images of 1998 left and right coronary arteries shown in *Figure 2D*. In 1998, she was admitted directly from her son’s wedding. The InterTak diagnostic score and probability in 1998 was the same as in 2014.

**Figure 3:**
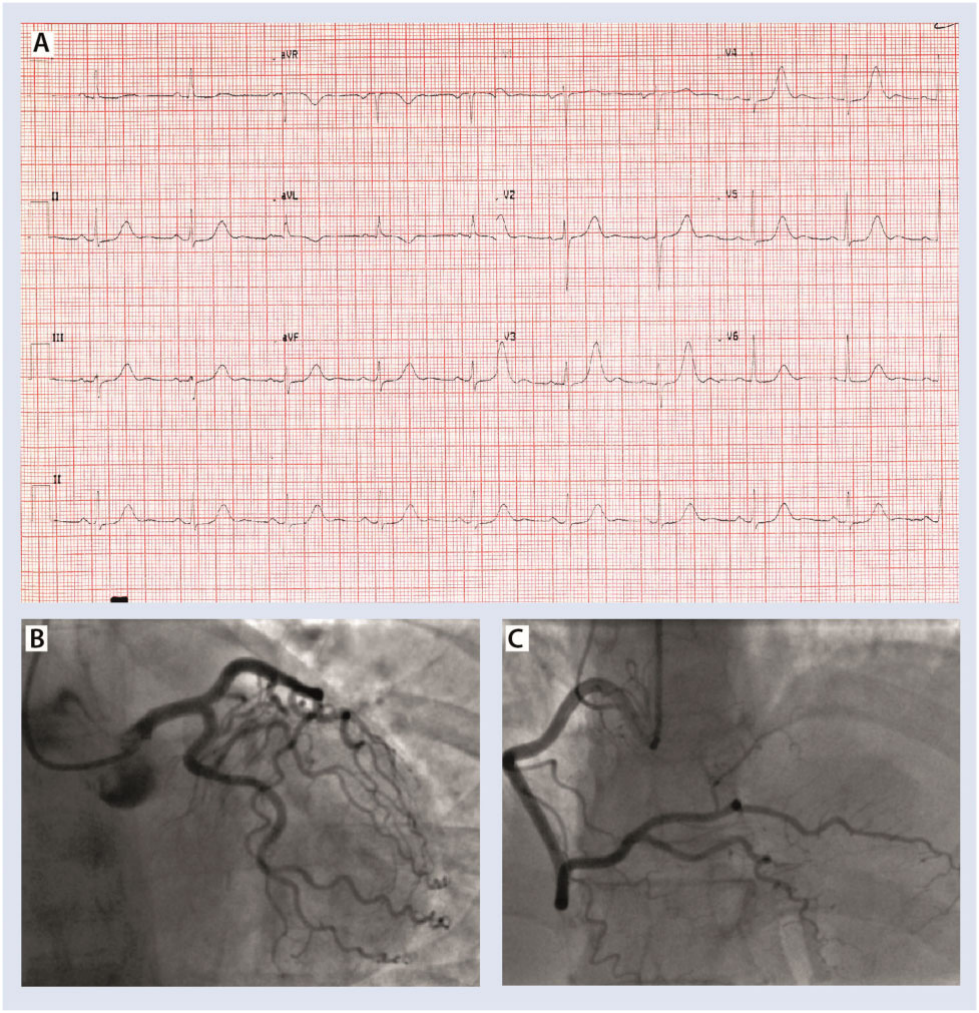
(*A*) Presenting electrocardiogram 2015, Qtc 560 ms. (*B* and *C*) Left and right coronary arteries, respectively. See also Supplementary material online, Movie M5: left coronary artery 2015; Supplementary material online, Movie M6: right coronary artery 2015.

Subsequently, she represented with central chest pain in 2015, on the background of being the main carer of her husband who had a stroke. Initial troponin was 75 times more than the upper limit of normal and the repeat 12 h troponin was 48 times the upper limit of normal contemporary range. Electrocardiogram on admission is shown in *Figure 3A*. Again, coronary arteries were normal as shown in *Figure 3B* and *C*. She had a left ventriculogram demonstrated probable mild area of hypokinesis in the inferior apical wall, supporting the diagnosis of apical Takotsubo. Her InterTak diagnostic score was 67, with a 79.8% probability of takotsubo.

**Figure 4:**
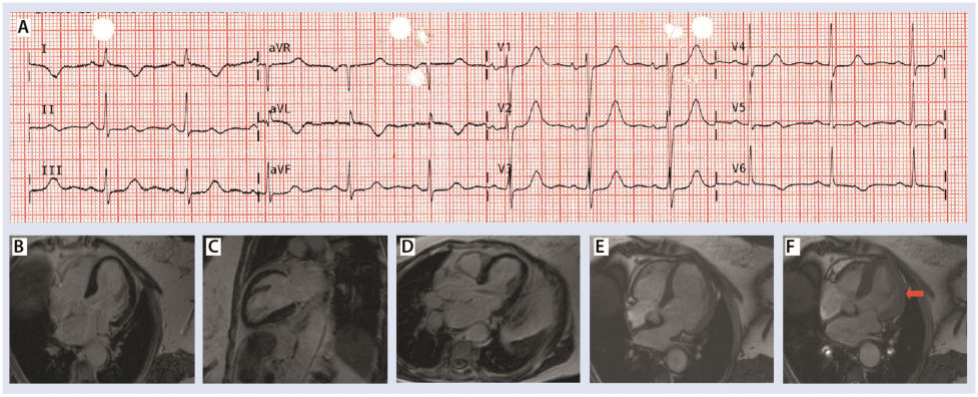
(*A*) Ambulance electrocardiogram 2018. (*B–D*) Focal phenotype of Takotsubo. (*E* and *F*) End-diastolic and end-systolic, respectively. Red arrow in *F* shows mid-cavity focal ballooning. Supplementary material online, Movie M7: four-chamber cardiac magnetic resonance cine demonstrating the focal lateral wall ballooning.

Further, in 2018, she had another episode of chest pain. Ambulance ECG is shown in *Figure 4A* with widespread T-wave inversion in the lateral leads and QTc of 527 ms. Initial troponin on admission was increased to 61 times the upper limit of normal contemporary range and repeat troponin 6 h later was again raised to 59 times the upper limit of normal contemporary range. InterTak diagnostic score was raised to 67, with a probability of 79.8%. Angiogram revealed normal coronary arteries and a focal phenotype of takotsubo cardiomyopathy best illustrated on cardiac MRI (*Figure 4B*–*D*) which once again ruled out any myocardial infarction. *Figure 4E* and *F* shows the end-diastolic and end-systolic phase, respectively. Red arrow in *Figure 4F* illustrates mid-cavity focal ballooning. A four-chamber cardiac magnetic resonance is shown in Supplementary material online, *Movie M7*. Once again she made a good recovery.

Clinical follow-up 3 months after the 2018 episode confirmed good clinical recovery, unremarkable clinical examination, and normal left ventricular ejection fraction with no regional wall motion abnormalities. Based on the local policy of not medicating these patients due to the lack of supporting evidence, she was discharged on no specific therapy.

## Discussion

Here, we present a case of most likely six recurrences of takotsubo cardiomyopathy spanning over 33 years. At least three presentations had different left ventricular morphological types (apical, apical-to-mid-cavity, and focal) some were triggered by obvious and declared stress, while some were not. Recurrence of Takotsubo Cardiomyopathy is a well-known reported long-term issue in the takotsubo cohort. The recurrence rate is ∼5–6% at 6 years.^3^^,^^4^

To date, early cardiac catheterization is the only definite way to distinguish between acute coronary syndrome and takotsubo. However, to better guide initial evaluation, the non-invasive novel InterTak diagnostic score has been proposed.^5^ It helps distinguish takotsubo from acute coronary syndrome with good sensitivity and specificity using seven variables each with an assigned score value.

Three episodes were potentially mislabelled as ‘myocardial infarction’ in the past, despite showing normal coronary arteries on each occasion and no evidence of myocardial injuries noted on subsequent cardiac magnetic resonance. The diagnosis of takotsubo was only discovered in 1991,^6^ hence it is understandable that this diagnosis was not recognized during the 1986 and 1988 episodes. The diagnosis of takotsubo could not be confirmed in 1998 due to the historic limitations with the lack of cardiac magnetic resonance and non-specific cardiac enzymes used.

Clinicians must retain increased awareness on the possibility of multiple recurrences within the same individual, their shifting morphological variants on presentation and the possibility of previous episodes which may have been mislabelled in the past.

We would like to highlight the need for more multicentre prospective clinical trials and registries of takotsubo cases to further explore the best management and follow-up strategy of similar cases.

## Lead author biography

**Figure:**
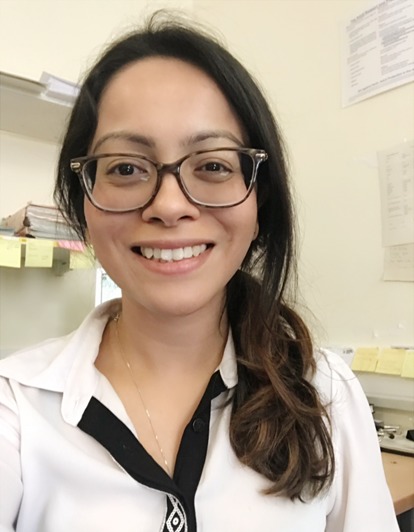


Dr Shekinah Chandy (MBChB Aberdeen) currently an Internal Medical Trainee Year 1 in Aberdeen Royal Infirmary, with a keen interest in cardiology and research.

## Supplementary material

Supplementary material is available at *European Heart Journal - Case Reports* online.

## Funding

This patient’s clinical investigation course was supported by the British Heart Foundation Project [PG/15/108/31928].

**Slide sets:** A fully edited slide set detailing this case and suitable for local presentation is available online as Supplementary data.

**Consent:** The author/s confirm that written consent for submission and publication of this case report including image(s) and associated text has been obtained from the patient in line with COPE guidance. 

**Conflict of interest:** none declared.

## Supplementary Material

ytz191_Supplementary_Data
