# Supplementary material for: Lifelong recurrent takotsubo cardiomyopathy: a case report
Source: Eur Heart J Case Rep. 2019 Oct 24;3(4):1–5. doi: 10.1093/ehjcr/ytz191 (PMC7042134; doi:10.1093/ehjcr/ytz191)
Supplement: ytz191_Supplementary_Data [file ytz191_supplementary_data.zip › ytz191-Suppl_data/Supplementary_Slide_Set.pptx]

## Slide 1
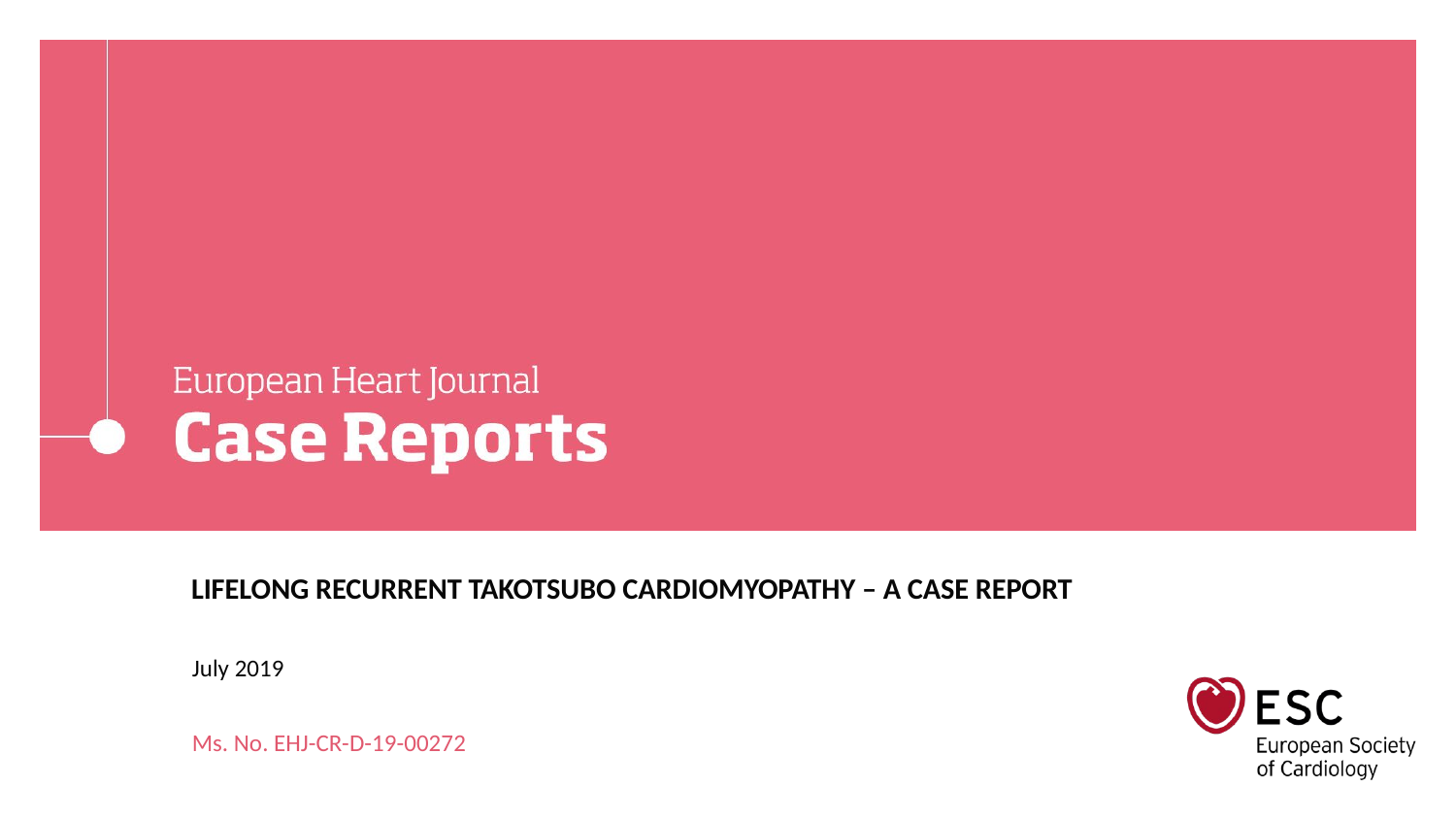

# LIFELONG RECURRENT TAKOTSUBO CARDIOMYOPATHY – A CASE REPORT
July 2019
Ms. No. EHJ-CR-D-19-00272

## Slide 2
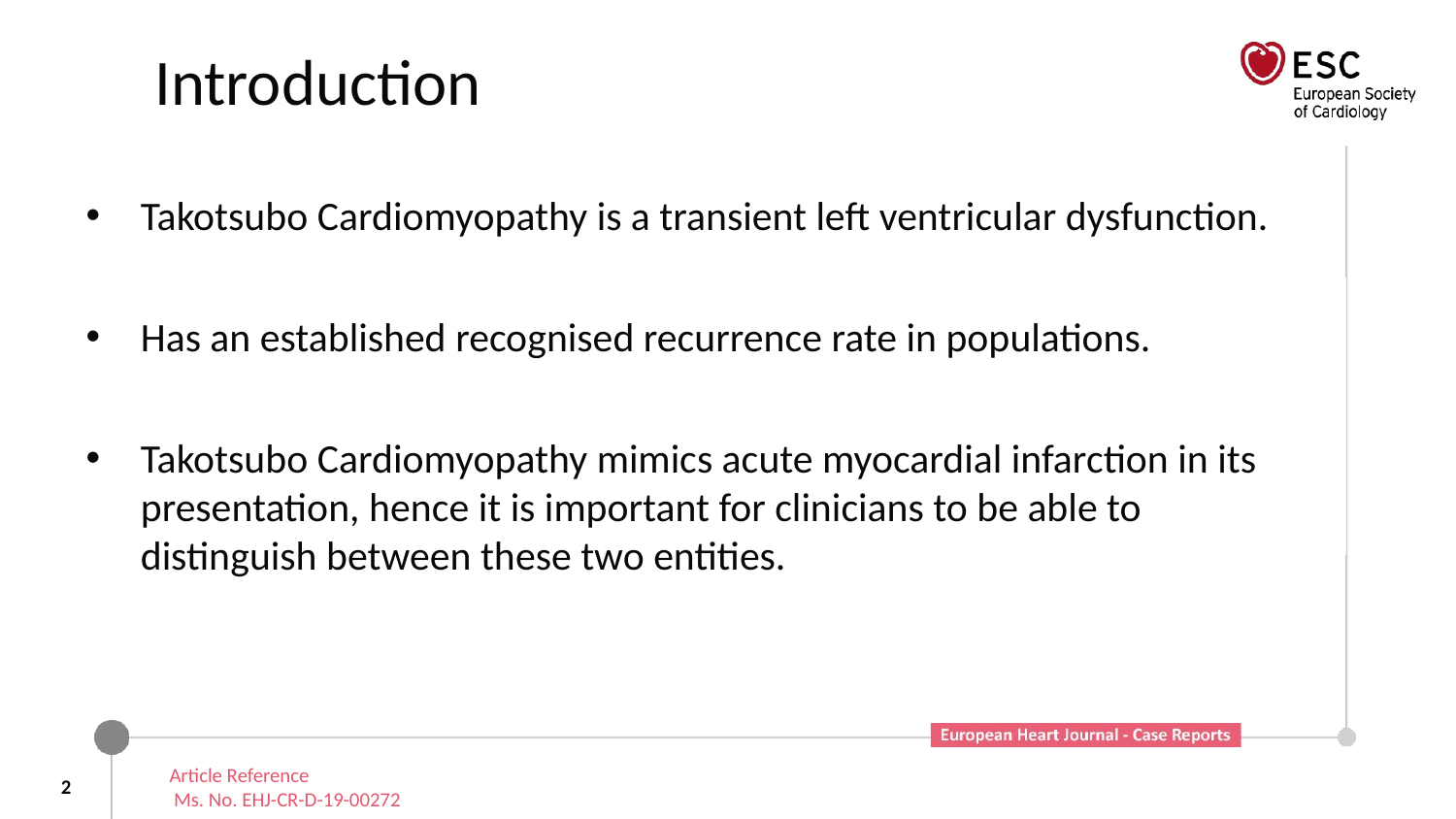

# Introduction
Takotsubo Cardiomyopathy is a transient left ventricular dysfunction.
Has an established recognised recurrence rate in populations.
Takotsubo Cardiomyopathy mimics acute myocardial infarction in its presentation, hence it is important for clinicians to be able to distinguish between these two entities.
2
Article Reference
 Ms. No. EHJ-CR-D-19-00272

## Slide 3
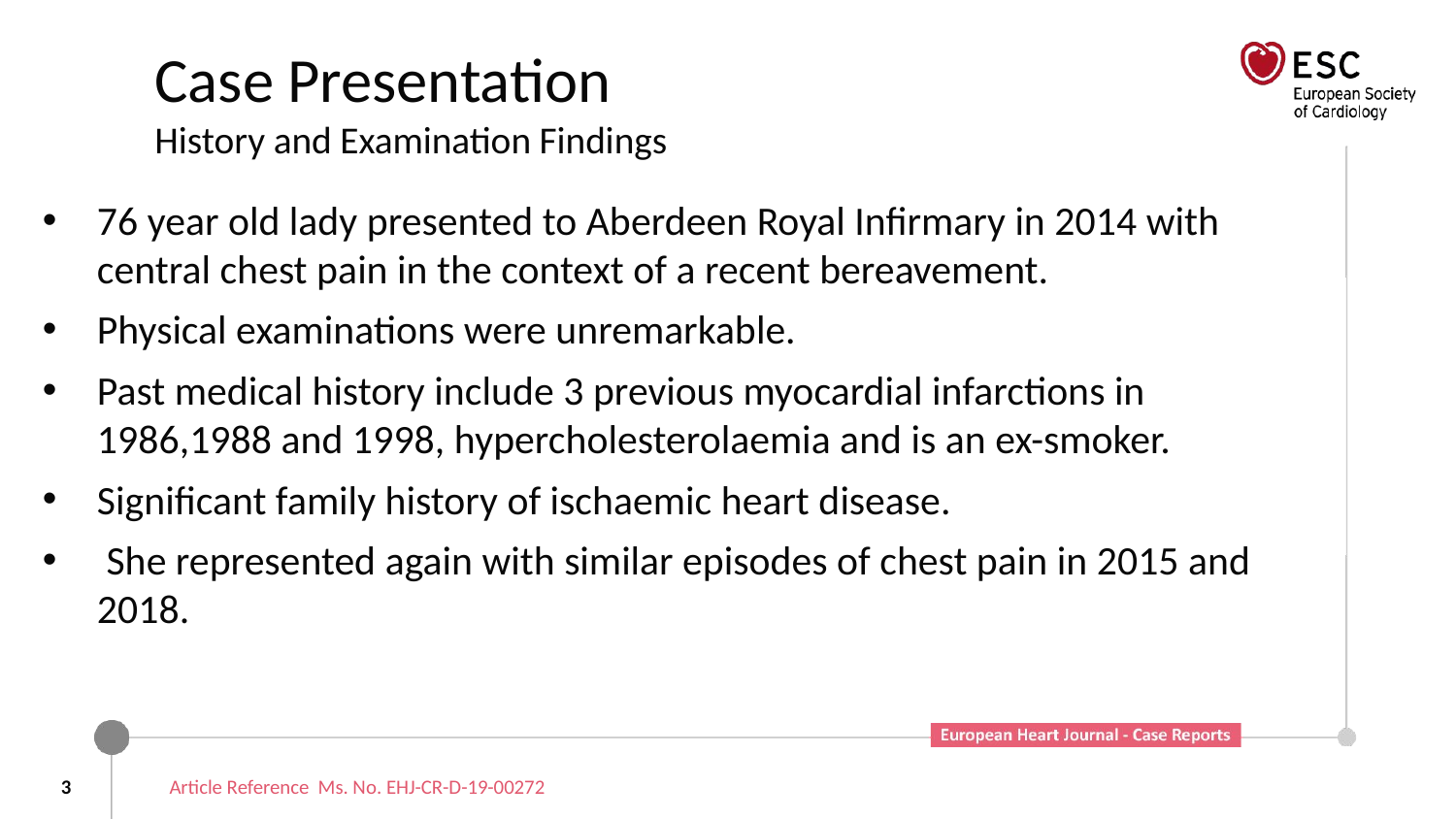

# Case Presentation History and Examination Findings
76 year old lady presented to Aberdeen Royal Infirmary in 2014 with central chest pain in the context of a recent bereavement.
Physical examinations were unremarkable.
Past medical history include 3 previous myocardial infarctions in 1986,1988 and 1998, hypercholesterolaemia and is an ex-smoker.
Significant family history of ischaemic heart disease.
 She represented again with similar episodes of chest pain in 2015 and 2018.
3
Article Reference Ms. No. EHJ-CR-D-19-00272

## Slide 4
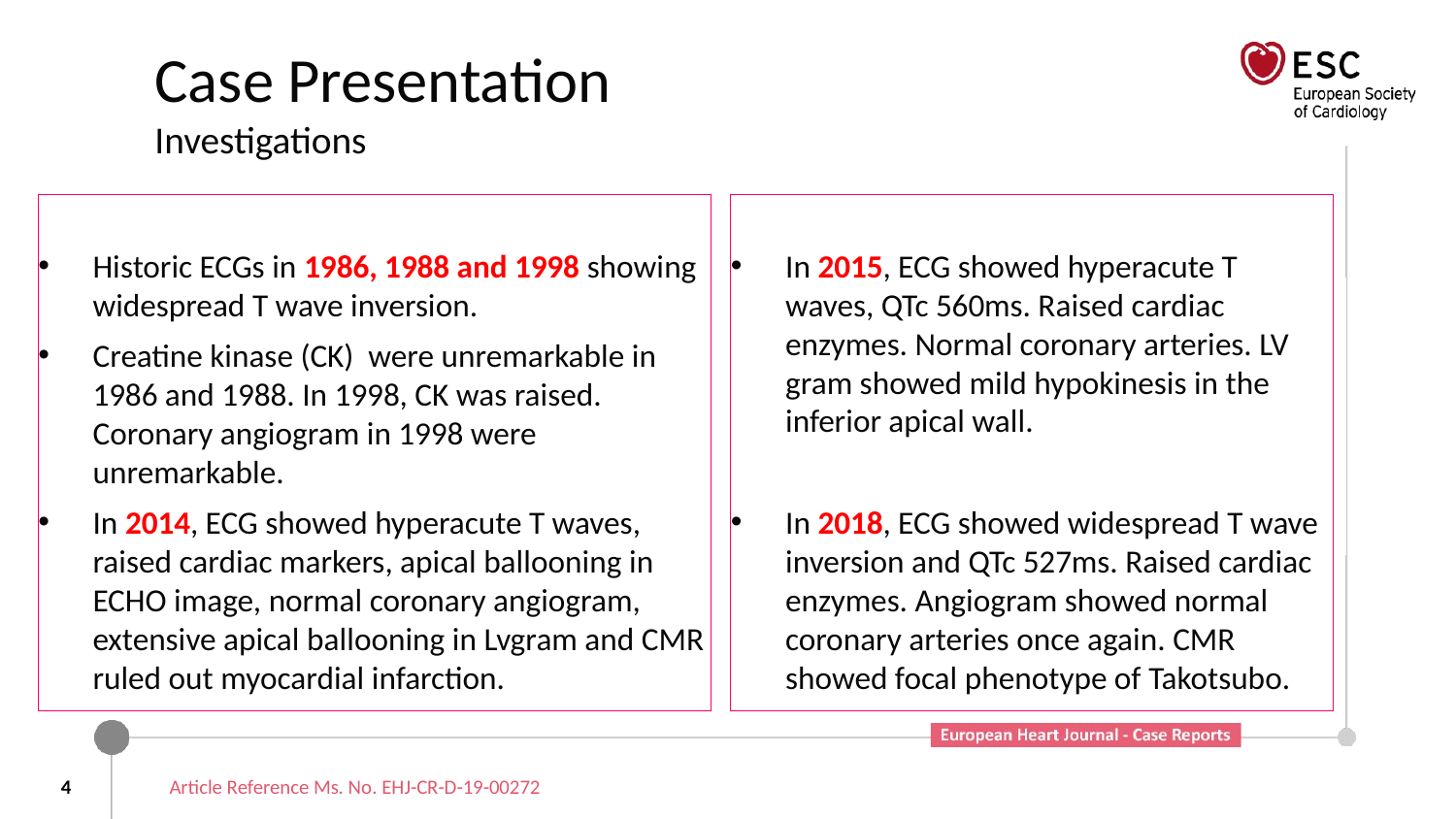

# Case Presentation Investigations
Historic ECGs in 1986, 1988 and 1998 showing widespread T wave inversion.
Creatine kinase (CK) were unremarkable in 1986 and 1988. In 1998, CK was raised. Coronary angiogram in 1998 were unremarkable.
In 2014, ECG showed hyperacute T waves, raised cardiac markers, apical ballooning in ECHO image, normal coronary angiogram, extensive apical ballooning in Lvgram and CMR ruled out myocardial infarction.
In 2015, ECG showed hyperacute T waves, QTc 560ms. Raised cardiac enzymes. Normal coronary arteries. LV gram showed mild hypokinesis in the inferior apical wall.
In 2018, ECG showed widespread T wave inversion and QTc 527ms. Raised cardiac enzymes. Angiogram showed normal coronary arteries once again. CMR showed focal phenotype of Takotsubo.
4
Article Reference Ms. No. EHJ-CR-D-19-00272

## Slide 5
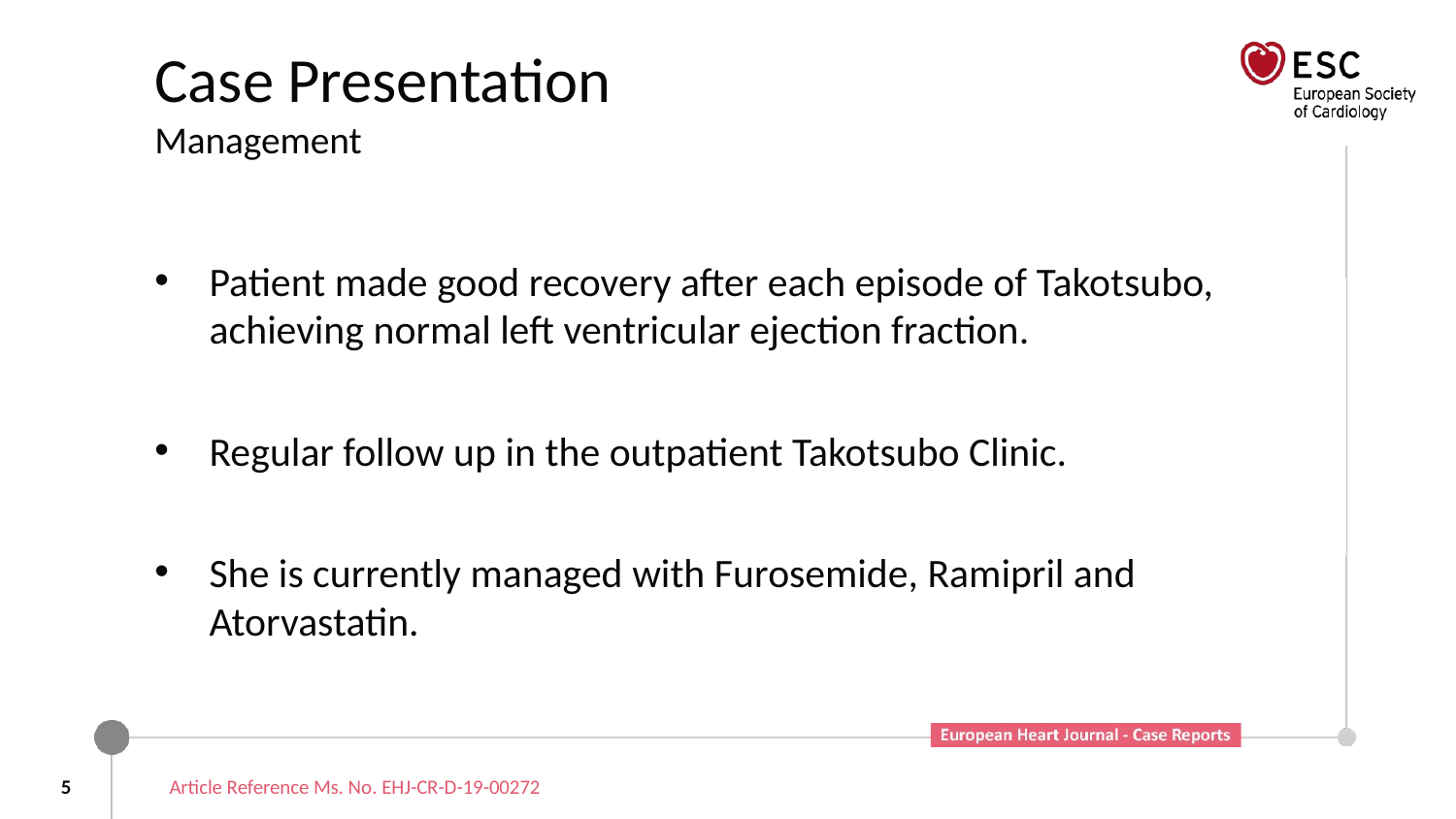

# Case Presentation Management
Patient made good recovery after each episode of Takotsubo, achieving normal left ventricular ejection fraction.
Regular follow up in the outpatient Takotsubo Clinic.
She is currently managed with Furosemide, Ramipril and Atorvastatin.
5
Article Reference Ms. No. EHJ-CR-D-19-00272

## Slide 6
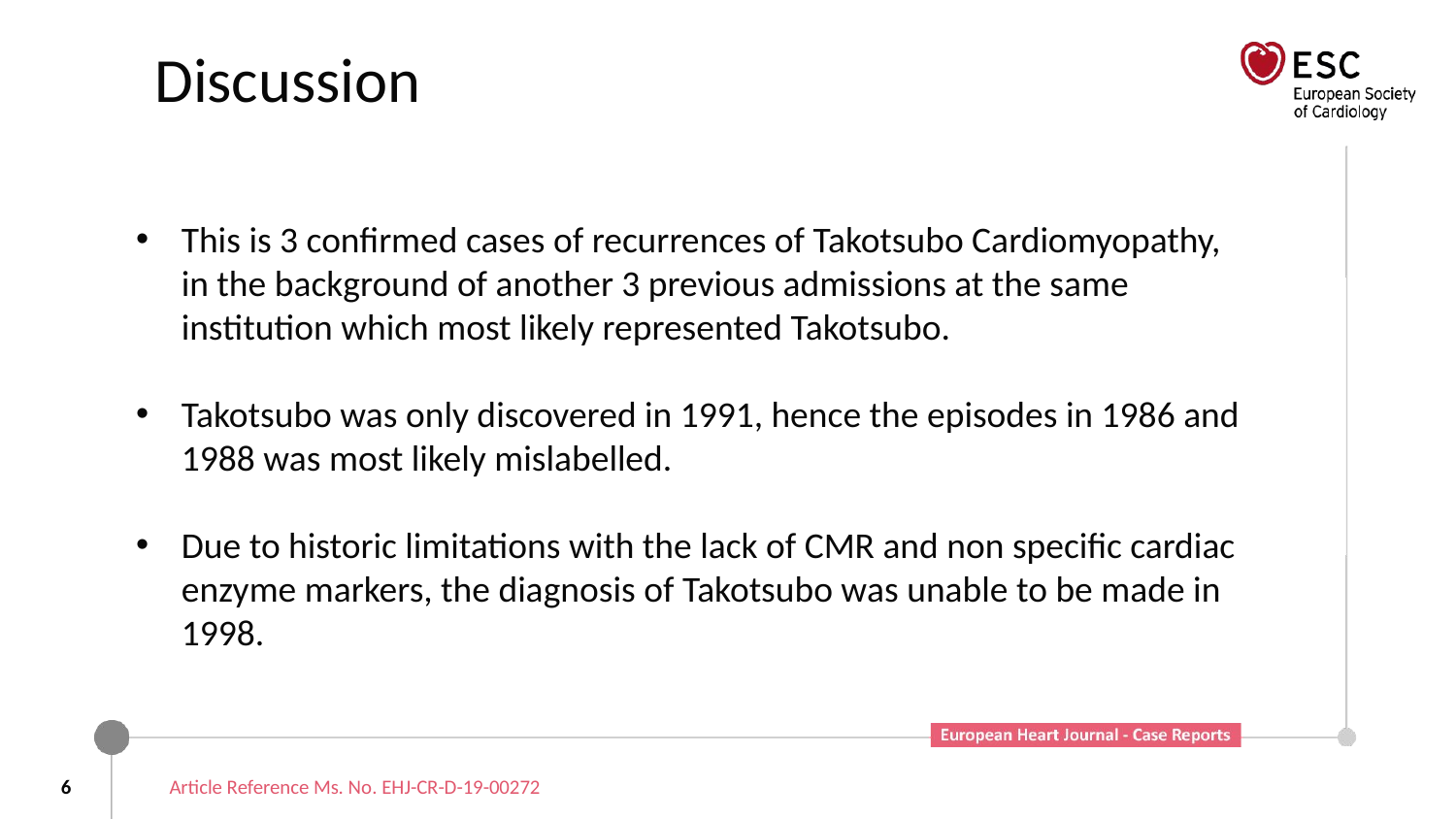

# Discussion
This is 3 confirmed cases of recurrences of Takotsubo Cardiomyopathy, in the background of another 3 previous admissions at the same institution which most likely represented Takotsubo.
Takotsubo was only discovered in 1991, hence the episodes in 1986 and 1988 was most likely mislabelled.
Due to historic limitations with the lack of CMR and non specific cardiac enzyme markers, the diagnosis of Takotsubo was unable to be made in 1998.
6
Article Reference Ms. No. EHJ-CR-D-19-00272

## Slide 7
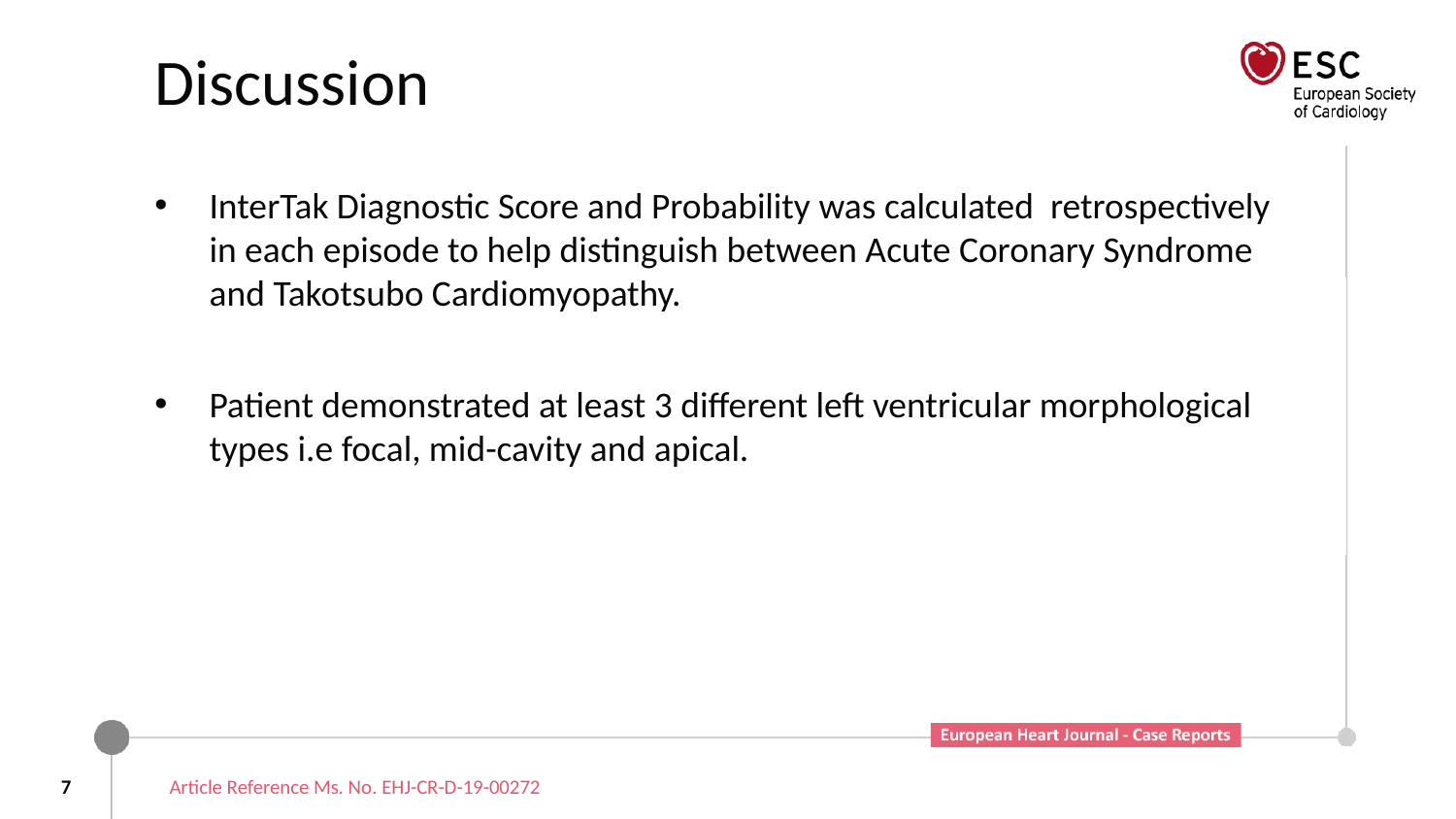

# Discussion
InterTak Diagnostic Score and Probability was calculated retrospectively in each episode to help distinguish between Acute Coronary Syndrome and Takotsubo Cardiomyopathy.
Patient demonstrated at least 3 different left ventricular morphological types i.e focal, mid-cavity and apical.
7
Article Reference Ms. No. EHJ-CR-D-19-00272

## Slide 8
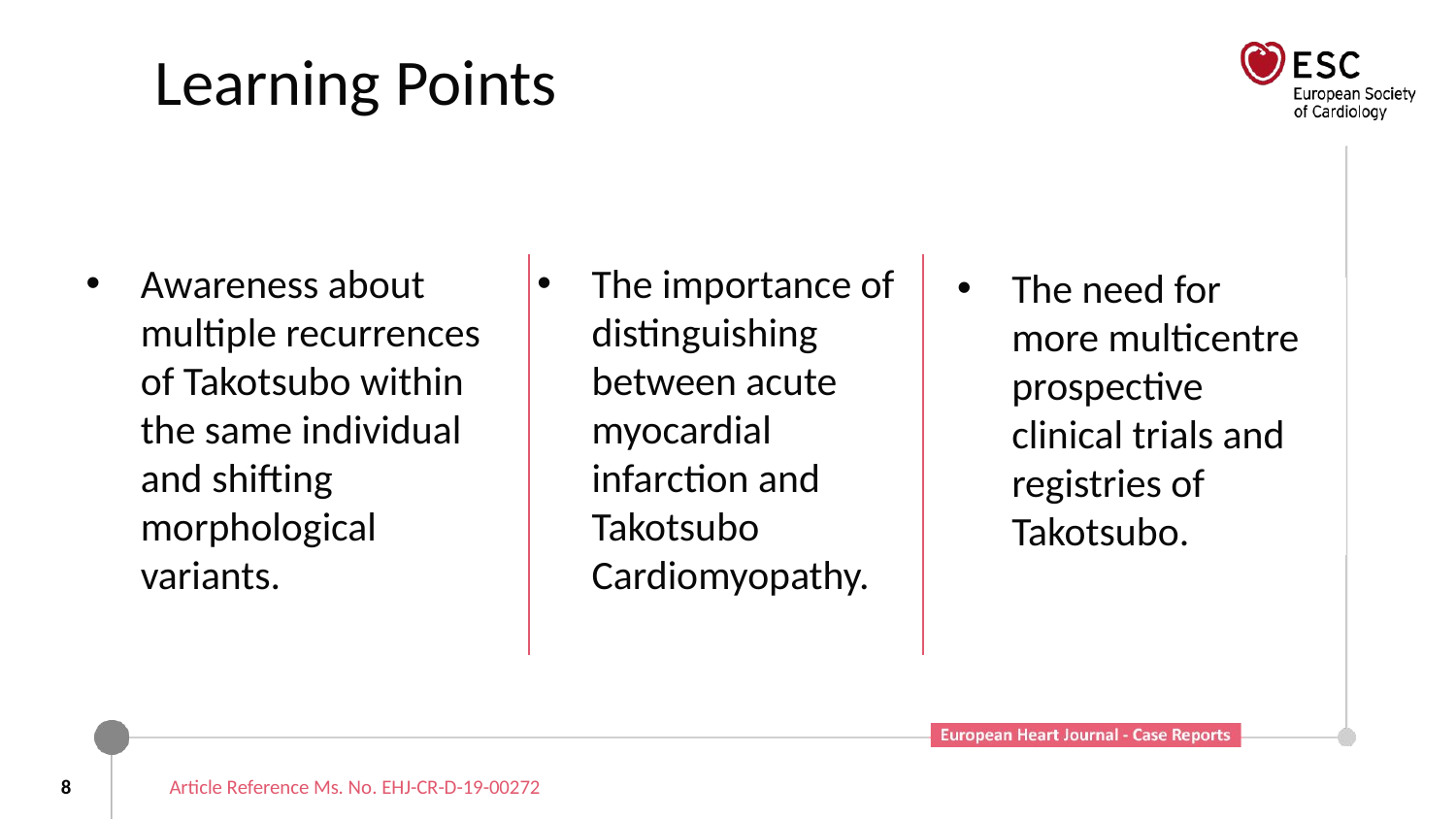

# Learning Points
Awareness about multiple recurrences of Takotsubo within the same individual and shifting morphological variants.
The importance of distinguishing between acute myocardial infarction and Takotsubo Cardiomyopathy.
The need for more multicentre prospective clinical trials and registries of Takotsubo.
8
Article Reference Ms. No. EHJ-CR-D-19-00272

## Slide 9
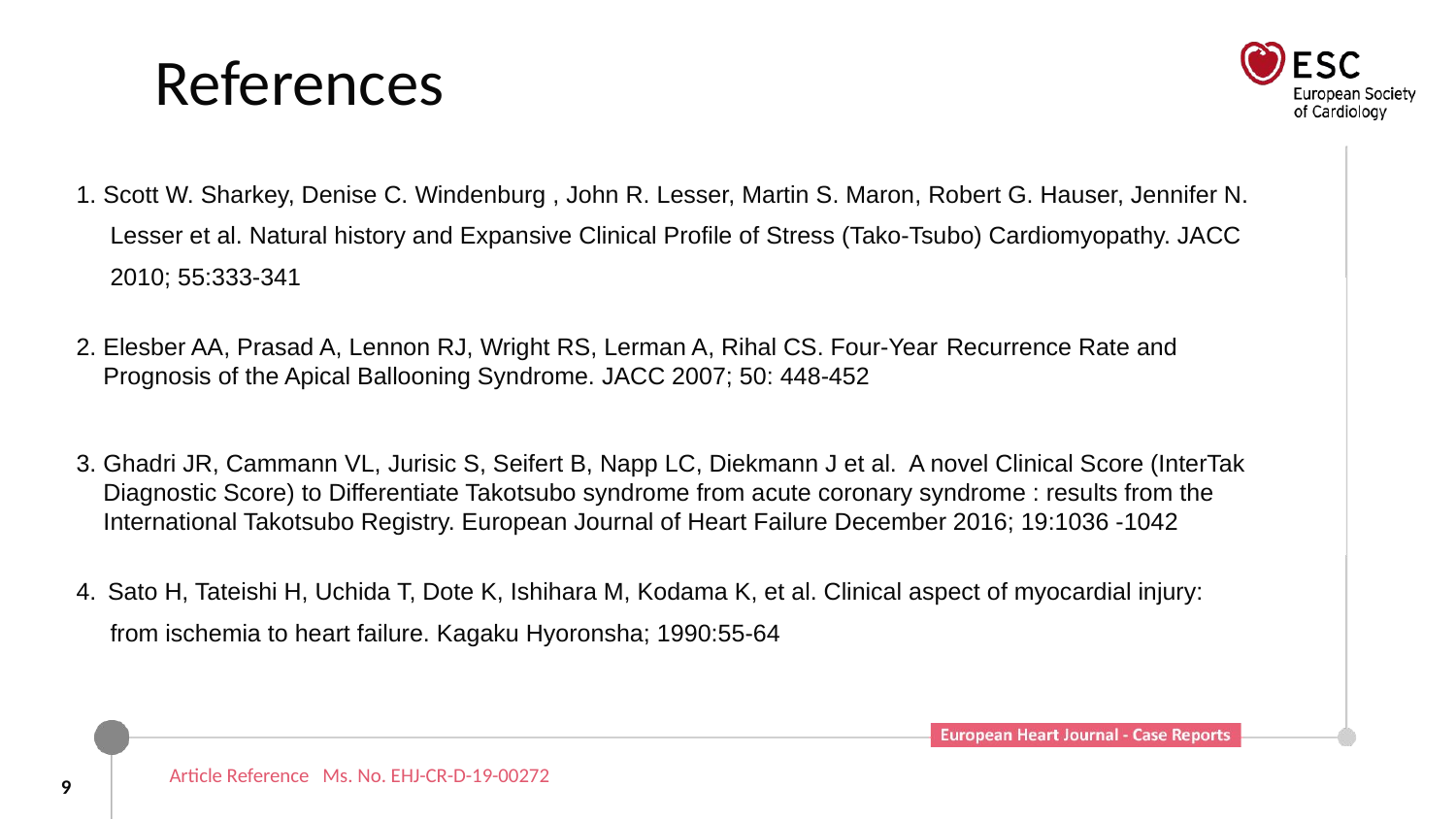

# References
1. Scott W. Sharkey, Denise C. Windenburg , John R. Lesser, Martin S. Maron, Robert G. Hauser, Jennifer N.
 Lesser et al. Natural history and Expansive Clinical Profile of Stress (Tako-Tsubo) Cardiomyopathy. JACC
 2010; 55:333-341
2. Elesber AA, Prasad A, Lennon RJ, Wright RS, Lerman A, Rihal CS. Four-Year Recurrence Rate and
 Prognosis of the Apical Ballooning Syndrome. JACC 2007; 50: 448-452
3. Ghadri JR, Cammann VL, Jurisic S, Seifert B, Napp LC, Diekmann J et al. A novel Clinical Score (InterTak
 Diagnostic Score) to Differentiate Takotsubo syndrome from acute coronary syndrome : results from the
 International Takotsubo Registry. European Journal of Heart Failure December 2016; 19:1036 -1042
4. Sato H, Tateishi H, Uchida T, Dote K, Ishihara M, Kodama K, et al. Clinical aspect of myocardial injury:
 from ischemia to heart failure. Kagaku Hyoronsha; 1990:55-64
9
Article Reference Ms. No. EHJ-CR-D-19-00272
